# Expression of Notch-1 and its clinical significance in different histological subtypes of human lung adenocarcinoma

**DOI:** 10.1186/1756-9966-32-84

**Published:** 2013-10-27

**Authors:** Jiayuan Huang, Haizhu Song, Biao Liu, Bo Yu, Rui Wang, Longbang Chen

**Affiliations:** 1 Department of Medical Oncology, Jinling Hospital, School of Medicine, Nanjing University, 315 Zhongshan East Road, Nanjing, Jiangsu 210002, PR China; 2 Department of Pathology, Jinling Hospital, School of Medicine, Nanjing University, 305 Zhongshan East Road, Nanjing 210002, China

**Keywords:** Lung adenocarcinoma, Notch-1, Immunohistochemistry, Histological subtypes, Prognosis

## Abstract

**Background:**

According to the International Multidisciplinary Classification of Lung Adenocarcinoma (LAD) by International Association for the Study of Lung Cancer/American Thoracic Society/European Respiratory Society (IASLC/ATS/ERS) in 2011, the diagnosis of LAD is changing from simple morphology into a comprehensive multidisciplinary classification. The aim of this study is to detect the expression of Notch-1 and analyze its clinicopathological or prognostic significance in different histological subtypes of Lung Adenocarcinomas (LADs).

**Methods:**

Western blot and Semi-quantitative Reverse transcription-polymerase chain reaction (RT-PCR) assays, as well as immunohisitochemistry, were performed to detect the expression of Notch-1 in LAD cells and tissue samples. Kaplan-Meier and multivariate Cox regression analyses were performed to evaluate the correlation of Notch-1 expression with clinicopathological factors and prognosis of LAD patients.

**Results:**

The expression level of Notch-1 protein in LAD cell lines or tissues was significantly lower than that in normal human bronchial epithelial cell line (16HBE) or nontumor tissues (*P* < 0.05). By statistical analyses, it was observed that negative Notch-1 expression was significantly associated with advanced clinical stage (*P* = 0.001) and lymph node metastasis (*P* = 0.026) in LAD patients. Also, the recurrence rate of Notch-1-positive group was higher than the Notch-1-negative group (*P* = 0.001), and patients with positive Notch-1 expression have a prolonged progression of overall survival (*P* = 0.033). More interestingly, the expression of Notch-1 protein was often observed to be negative in solid predominant adenocarcinoma (SPA) tissues, but highly expressed in papillary predominant adenocarcinoma (PPA) and micropapillary predominant adenocarcinoma (MPA) tissues. Kaplan-Meier survival analysis showed that patients with positive Notch-1 expression had a prolonged progression of overall survival compared with those with negative Notch-1 expression (*P* = 0.033). The median survival time of Notch-1-positive or negative patients was 64.6 months (95% CI: 31.497-97.703 months) or 36.0 months (95% CI: 12.132-59.868 months).

**Conclusions:**

Notch-1 could be used as a predictable biomarker to be detected in different pathological and histological subtypes in LAD for diagnosis or prognosis.

## Background

Lung cancer is the most common cancer and the leading cause of cancer deaths around the world [1]. Although prognosis of patients can be improved through effective treatment, the 5-year survival rate of patients with advanced lung cancer is only 10%-15% [2]. Non-small cell lung cancer (NSCLC) accounts for 70%-80% in lung cancer, and among them, lung adenocarcinoma (LAD) accounting for almost half of lung cancers, was one of the most common histologic subtype. Patients with LAD had rapid disease progression, and recurrence ratio was high even after surgery. Despite the recent progresses made in diagnostic techniques and combined treatments for this disease, the overall 5-year survival rate of LAD patients was still less than 30% [3-5]. Thus, a better understanding the molecular mechanisms involved in the pathogenesis of LAD will be helpful for the development of better prognostic markers and novel therapeutic targets to improve clinical treatment of LAD patients.

Recently, more and more studies gradually reveal that dysregulation of the Notch signaling pathway plays a pivotal role in the pathogenesis of many human malignancies. Notch-1, one of the key receptor in the Notch signaling pathway, encodes an important member of Notch family proteins [6]. Increasing evidences have shown that Notch-1 is involved in the regulation of tumor cell growth, proliferation, apoptosis, metastasis and chemo- or radioresistance. Notch-1 was either reported as an oncogene [7] in some solid tumors, or reported as a tumor suppressor in other tumors [8]. The two different viewpoints were usually resulted from different types of tumors or different stages of tumors. For example, some scholars showed that Notch-1 could be activated to inhibit growth of small cell lung cancer cells, while it was also found to promote growth of NSCLC cells [9]. Depicted on the International Multidisciplinary Classification of LAD by International Association for the Study of Lung Cancer/American Thoracic Society/European Respiratory Society (IASLC/ATS/ERS) published in 2011 [10], the standard of diagnosis is refining and adjusting to a comprehensive multidisciplinary classification. Thus, it is needed to detect the expression of Notch-1 protein and analyze its clincopathological or prognostic significance in different histological subtypes of human LADs.

In the present study, Western blot assay was performed to detect the expression of Notch-1 protein in LAD cell lines and tissue samples. Also, immunohistochemistry assay was performed to detect the expression of Notch-1 protein in 101 cases of LAD tissues with different histological subtypes. Then, the correlations of Notch-1 protein expression with clinicopathological factors of LAD patients were statistically analyzed. Additionally, the relationships of Notch-1 with histological subtypes and survival prognosis of LAD patients were investigated.

## Materials and methods

### Patients and tissue samples

A total of 101 LAD tissues in Thoracic Surgery Department of Jinling Hospital from January 2005 to December 2007 were collected. All patients have signed the Informed Consent. Every patient’s basic clinical records were reviewed, including age, gender, operation time, surgical site and related pathological data. The morphological classification and metastasis judgment were determined by two senior pathologists. Cases were followed up by the form of telephone, patients’ overall survival (OS) time were defined from surgery to the date of death or the latest follow-up. The deadline of follow-up was September-15, 2012. This Research was granted scientific and ethic approval by The Ethics Committee of Jinling Hospital.

### Cell lines

A normal human bronchial epithelial cell line (16HBE) and three human LAD cell lines (SPC-A1, A549 and H1299) were all purchased from the American type culture collection (ATCC). Tumor cells were expanded in RPMI 1640 medium supplemented with 10% fetal bovine serum (FBS) and ampicillin and streptomycin at 37°C in a humidified atmosphere with 5% CO2, and 16HBE cell line was maintained in Dulbecco’s Modified Eagle’s Medium (DMEM) with 10% FBS and ampicillin and streptomycin in the same environment.

### Semi-quantitative reverse transcription-polymerase chain reaction (RT-PCR)

Total RNA were extracted from different cultured cell lines by TRIzol reagent (Invitrogen) following the manufacturer’s instructions. 1 ug RNA from each cell was provided to cDNA synthesis using oligo-dT as a primer by PrimeScript™ RT reagent Kit (Takara). The procedure of Reverse transcription reaction was 37°C for 15 min, followed by 85°C for 5 seconds. The primers used for amplification of Notch-1 were designed as followed: Notch-1 sense, forward 5'-CCGTCATCTCCGACTTCATCT-3′and reverse 5'-GTGTCTCCTCCCTGTTGTTCTG-3′. Glyceraldehyde-3-phosphate dehydrogenase (GAPDH) was chosen to be inner control, forward sense 5’-GCACCGTCAAGGCTGAGAAC-3’ and reverse 5’-TGGTGAAGACGCCAGTGGA-3’. PCR reactions were achieved in the total volume of 25 ul mixture, including 9.5 μl of H2O, 1 μl of forward and reverse primers, 1 μl of cDNA and 12.5 μl of 2X SYBR Green PCR Master Mix. The procedures of PCR were initial denaturation at 95°C for 3 min, then 35 cycles of duraturation at 94°C for 40 sec, annealing at 58°C for 40 sec, elongation at 72°C for 90 s. At last elongation sufficiently for 10 min. The amplified products were captured by electrophoresis with 1.5% agarose gel.

### Western blot analysis

The fresh tissues were all random selected from Chest surgery department of Jinling Hospital. All the cells and tissue samples were lysed in ice-cold buffer containing RIPA lysate with protease inhibitor cocktail and 1 mmol/L Phenylmethanesulfonyl fluoride (PMSF) for about 20 min. Proteins were fractionated by 4%-8% SDS- polyacrylamide gel electrophoresis (SDS-PAGE), then followed by transferred to a polyvinylidene fluoride membrane, blocked by 5% non-fat milk with Tris-buffered salne. All blots were probed with primary antibody rabbit anti-human Notch-1 (1:1000 dilution; Val1744; Cell signaling technology), while rabbit anti-human β-actin (1:1000 dilution; 13E5; Cell signaling technology) was used as control. The membrane subsequently incubated with horseradish peroxidase (HRP)-links second antibodys after 4°C overnight. Finally, result was detected by ECL regent.

### Immunohistochemistry

All specimens were fixed in 4% formalin and embedded into wax blocks after surgery. The slides were treated with poly-lysine to preventing tissue loss. 3–4 μm thick consecutive paraffin sections were cut from each case and stained with hematoxylin and eosin (H&E) and immunohistochemical analysis by Maxvision. Routinely deparaffinized in xylene and decreasing concentrations of ethanol to water. Antigen was fixed by high temperature and pressure with citrate buffer solution (Maixin_bio MVS-0066). Each section was added 100 μl 3% H_2_O_2_ to block endogenous peroxidase activity in room temperature for 20 minutes. After washing by phosphate buffer solution (PBS, Maixin_bio PBS-0060/0061), 100 μl primary antibody (Santa cruze SC-6014, Notch-1) were incubated at 4°C overnight. And then followed by polink-2 plus Polymer HRP detection system for Goat Primary antibody (ZSGB-BIO, PV-9003), used reagent 1 100 μl each section at room temperature for 20 minutes, later washed off, reagent 2 was the same operation. Afterwards, diaminobenzidine (DAB, biogenex, HK1240411) was used as the color reagent before slides were counterstained with hematoxylin, then dehydrated step by step by using descending concertrations of ethanol, cleared with xylene, mounted with neutral gum. Simultaneously, using PBS (0.01 mol/L, PH = 7.4) instead of primary antibody as blank control.

All the sides were independently assessed by two pathologists. The immunostained results of Notch-1 protein were semi-quantitated according to the criteria from published literatures [2,11]. Each section randomly selected 5 high-power fields, positive cells represented by the percentage of totally number of similar cells. Details were as follows: 0 point for less than 5% positive cells; 1 for 5%-25% positive cells; 2 for 26%-50% positive cells; 3 for 51%-75% positive cells; 4 for more than 76% positive cells. The staining intensity was scored on a scale as weak, moderate or strong. 0 point for no stained; 1 for low stained (pale yellow); 2 for moderate stained (brown); 3 for strong stained (tan). After added the two scores, <3 was defined as negative, ≥3 was positive.

### Statistical analysis

The statistical analyses were performed using software SPSS version 17.0 (SPSS Inc, Chicago). Individual clinical information and pathological characteristics were summarized using descriptive statistics. Qualitative data were determined a possible clear correlation analysis by chi-square test or Fisher’s exact test if the number was less than 5. Survival time was measured from the date of surgery to the latest follow-up or the date of death. Univariate analysis, including Survival analysis, was estimated by Kaplan-Meier method. Log-rank test was used for comparison of survival rate. Cox proportional hazards regression model was used for multivariate analysis. *P* < 0.05 was considered to demonstrate statistical significance.

## Results

### Notch-1 expression in LAD cell lines or tissues

**Figure 1:**
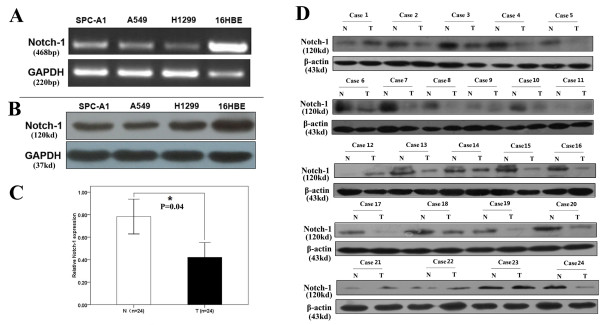
**Expression of Notch-1 in LAD Cell lines and tissues.** Semi-quantitative Reverse transcription-polymerase chain reaction **(A)** and Western Blot **(B)** were used to detect expression of Notch-1 in different cells of lung adenocarcinoma. Brochial epithelial cell was used as control. Weaker expression of Notch-1 was observed in tumor cells. Then, Notch-1 Protein in 24 tissues from surgery which diagnosed as lung adenocarcinoma were detected by Western Blot **(C and D)**. Each adjacent tissue from the same patient was used as control. Most of weaker performance was observed in tumor ones (*P* = 0.04).

First, nuclear acid detection and Western blot assays were performed to detect the expression of Notch-1 in a normal human bronchial epithelial cell line (16HBE) and three human LAD cell lines (SPC-A1, A549 and H1299). As shown in Figure 1A and B, the relative expression levels of Notch-1 mRNA and protein in LAD cell lines were significantly lower than those in normal human bronchial epithelial cell line. Next, the expression of Notch-1 in 24 postoperative cases of randomly selected LAD tissues and corresponding nontumor tissues was detected. As shown in Figure 1C and D, the mean expression level of Notch-1 mRNA and protein in LAD tissues were significantly lower than those in corresponding nontumor tissues (*P* = 0.04). These data indicated that downregulation of Notch-1 might play critical roles in LAD development.

### Clinicopathological variables of patients

Demographic, pathological and clinical variables were collected as below. It contained 64 male and 37 female. 50 patients were below 60-year-old. The age of patients at the time of diagnosis were ranging from 25 to 81-year-old, the median was 58.83-year-old. 37 patients had a smoking history in this 101 LAD cases. All the patients had undergone curative resection of LAD, 58 tumors (57.4%) were located in right, 43 ones (42.6%) were left. 39 cases (46.98%) relapsed, and 57 cases (56.4%) had lymph node metastasis. According to the Union for International Cancer Control (UICC) TNM classification of Malignant Tumours 7th edition [12] , there were 45 patients in stage I, 32 patients in stage II, 20 patients in stage III, 4 patients in stage IV. Meanwhile, 44 cases were poorly-differentiated, 47 were moderate-differentiated, and 10 were well-differentiated. By histological analyses [10], 41 patients were acinar predominant adenocarcinoma (APA), 20 were papillary predominant adenocarcinoma (PPA), 25 were solid predominant adenocarcinoma (SPA) with mucin production, 15 were other types including lepidic predominant adenocarcinoma (LPA), micropapillary predominant adenocarcinoma (MPA) and adenosquamous carcinoma. General clinical information of patients was shown in Table 1.

**Table 1:** **Relationship between expression of Notch**-**1 and clinicopathologic characteristics of LAD patients**

**Characteristics**			**Notch-1**			
		**n**	**(+)**	**(-)**	**x**^**2**^	***P***
Gender					0.123	0.726
	Male	64	22	42		
	Female	37	14	23		
Age (year)					0.240	0.624
	≥	51	17	34		
	<	50	19	31		
Histology					9.721	0.021*
	APA	44	17	27		
	PPA	20	9	11		
	SPA	25	3	22		
	Others	12	7	5		
Clinical stage					14.028	0.001**
	I	45	25	20		
	II/III/V	56	11	45		
Differentiation					3.850	0.05*
	Poor	44	11	33		
	moderate	47	21	26		
	well	10	4	6		
Lymph node Metastasis					4.963	0.026*
	N_0_	44	21	23		
	N_1-3_	57	15	42		
Recurrence					8.241	0.004**
	present	39	10	29		
	absent	44	25	19		
Smoking history					3.261	0.071
	Non-smoker	64	27	37		
	smoker	37	9	28		
Tumor location					0.08	0.777
	Right	58	20	38		
	Left	43	16	27		
Survival analysis					3.946	0.047*
	Death	45	14	31		
	Live	38	20	18		
	Disconnect	18	2	16		

*Abbreviation*: *APA* acinar predominant adenocarcinoma, *PPA* papillary predominant adenocarcinoma, *SPA* solid predominant adenocarcinoma, (+) positive; (-) negative. *P < 0.05, **P < 0.01.

### Immunostaining of Notch-1 protein in LAD tissues

**Figure 2:**
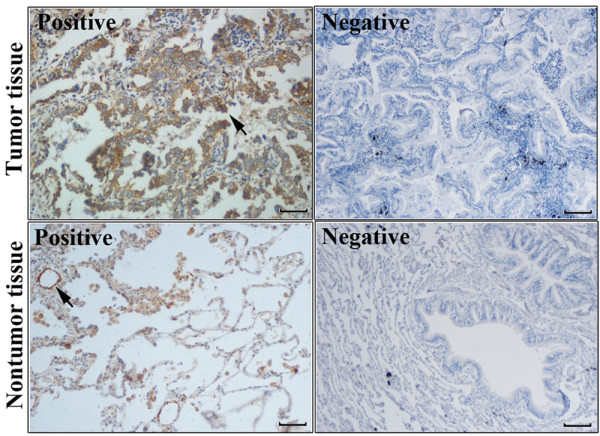
**The positive and negative expression of Notch-1 was detected in lung adenocarcinoma specimens.** It was not only in tumors but also in adjacent alveolar and brochial epithelial tissues. Black arrowheads indicated positive staining. Scale bar: 100 um.

**Figure 3:**
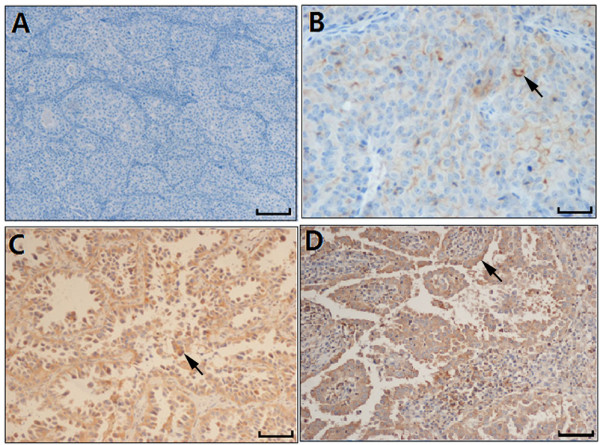
**Evaluation of Notch-1 IHC staining intensity. (A)**: no staining, 0; **(B)**: weak staining (pale yellow), 1+; **(C)**: moderate staining(brown), 2+; **(D)**: strong staining (tan), 3+. The sections which pointed with black arrows were considered as positve area. Scale bar: 100 um.

**Figure 4:**
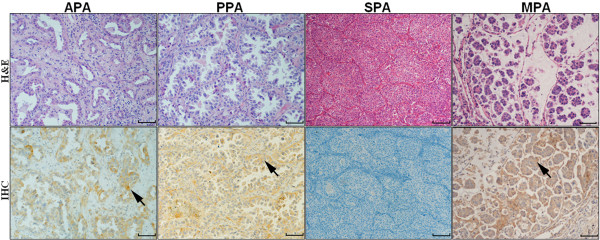
**Expression of Notch-1 in different histopathological subtypes of lung adenocarcinoma.** 17 APA patients (38.6%), 9 PPA patients (45.0%) and 7 other subtypes of patients (58.3%) were confirmed as positive (arrows), most SPA patients were confirmed as negative (*P* = 0.021), suggesting that immunostaining of Notch-1 in LAD tissues could be helpful for differentiating SPA from other histological subtypes. Scale bar = 100 um.

Immunohistochemistry was performed to detect the expression of Notch-1 protein in 101 cases of LAD tissues. As shown in Figure 2 and Figure 3, the positive Notch-1 protein was predominantly located in the cell membrane and (or) cytoplasmic, especially tumor cells. Brown granular staining was deemed as positive performance (black arrowheads). In 101 cases of LAD specimens, 36 (35.6%) cases were positive for Notch-1. Men were accounted for 22 patients (61.1%) of the positive group, whereas women were accounted for 14 patients (39.9%). 17 APA patients (38.6%), 9 PPA patients (45.0%) and 7 other subtypes of patients (58.3%) were confirmed as positive, but only 3 SPA patients (12.0%) was were confirmed as positive (*P* = 0.021; Figure 4), suggesting that immunostaining of Notch-1 in LAD tissues could be helpful for differentiating SPA from other histological subtypes.

### Correlation between Notch-1 expression and clinicopathological factors of LAD patients

The correlations of Notch-1 expression and clinicopathological factors of LAD patients were shown in Table 1. The difference by statistical analyses indicated that both clinical stages (*P* = 0.001) and recurrence of LAD patients (*P* = 0.004) were aware of predominant relevance with status of Notch-1 expression. Meanwhile, expression of Notch-1 was also found to be significantly correlated with histological subtypes (*P* = 0.021), tumor differentiation (*P* = 0.05), lymph node metastasis (*P* = 0.026). The positive ratio of Notch-1 protein expression in tissues from LAD patients with clinical stage I was significantly higher than that in tissues from patients with other clinical stages (II + III + IV). Also, tumors from LAD patients with positive Notch-1 expression showed better differentiation than those from patients with negative Notch-1 expression. Furthermore, the expression of Notch-1 protein was observed to be closely correlated with the survival endings of LAD patients (*P* = 0.047), and patients with positive Notch-1 expression had better survival endings than those with negative Notch-1 expression.

### Follow-up visit and prognostic factors analysis

**Figure 5:**
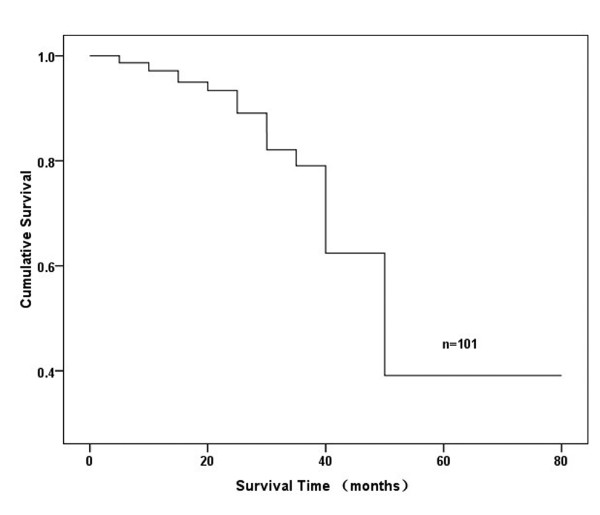
**The overall survival curve of patients with lung adenocarcinoma was done by life-tables.** During the time of follow-up, 45 patients (44.6%) were dead, 38 (37.6%) patients were alive, and 18 (17.8%) patients were lost. The mean 5-year survival rate of all patients was approximately 40%.

**Figure 6:**
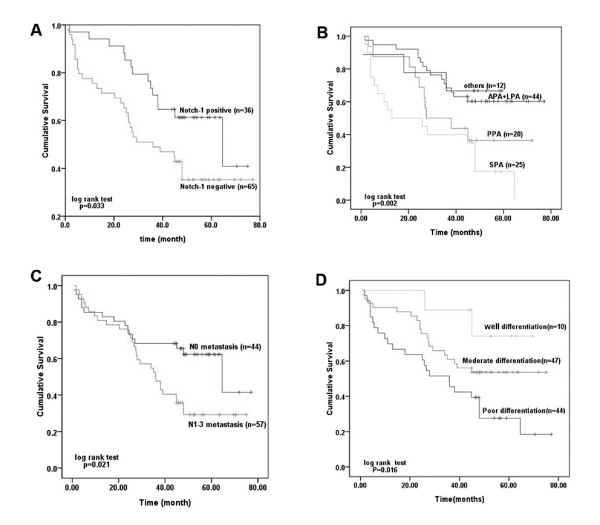
**Relationship between survival prognosis and related factors. (A)**: The correlation of Notch-1 expression and overall survival (OS) in Lung adenocarcinoma patients. Patients with high Notch-1 expression had a prolong OS (The median survival time was 64.6 months (95% CI: 31.497-97.703) versus 36.0 months (95% CI: 12.132-59.868), *P* = 0.033); **(B)**: The overall survival curves of different subtypes of lung adenocarcinoma. (*P* = 0.002); **(C, D)**: The overall survival curves of metastasis (*P* =0.021) and differentiation (*P* = 0.016).

In patients who were enrolled, the follow-up time was from 0.7 to 77.1 months, the average was 38.1 months. During the time of follow-up, 45 patients (44.6%) were dead, 38 (37.6%) patients were alive, and 18 (17.8%) patients were lost. The mean 5-year survival rate of all patients was approximately 40%, and the total survival curve was performed by life tables and shown in Figure 5. Notch-1 positive and negative groups exhibited differences in survival curves which were shown in Figure 6A. The median survival time of Notch-1-positive group was 64.6 months (95% CI: 31.497-97.703 months), but that of the negative group was only 36.0 months (95% CI: 12.132-59.868 months). The five-year survival rate of Notch-1-postive group (40.9%) was higher than that of Notch-1-negative group (35.3%), and statistical significance was exhibited (*P* = 0.033). Also, patients with different histological types showed different prognosis (Figure 6B), and it was found that patients with SPA showed worse survival than those with PPA, APA, LPA and others (*P* = 0.002). At the same time, we also showed that patients with no lymph node metastasis (N_0_) had better survival than those with lymph node metastasis (N_1_ + N_2_ + N_3_) (*P* = 0.021; Figure 6C). In addition, it could be observed that patients with well tumor differentiation had better survival than those with moderate or poor tumor differentiation (*P* = 0.016; Figure 6D).

For log-rank univariate analysis, the clinicopathological parameters such as age, gender, histological phenotype and others were included. As shown in Table 2, the status of Notch-1 expression, along with histological phenotype, lymph node metastasis and tumor differentiation, were found to be significantly associated with survival of LAD patients (*P* = 0.033, 0.002, 0.021 and 0.016, respectively). For further investigation, we analyzed the prognostic factors mentioned above by a multivariate Cox regression model (Table 2). The results indicated that only tumor differentiation was observed to an independent prognostic factor for LAD patients (*P* = 0.005). Although the status of Notch-1 was not an independent prognostic factor (*P* = 0.052), LAD patients with positive Notch-1 expression could show survival advantage.

**Table 2:** Results of univariate and multivariate Cox regression analysis of prognostic factors in LAD patients

**Variables**	**Univariate analysis**	**Multivariate analysis**		
	***P*** **value**	**RR**	**95% CI**	***P*** **value**
Age (≥60/<60)	0.149	1.009	0.98-1.04	0.579
Gender (Male/Female)	0.627	2.011	0.86-4.71	0.108
Clinical stage (I/II + III + IV)	0.214	0.467	0.11-2.14	0.328
Tumor localization (Left/Right)	0.268	1.083	0.57-2.07	0.809
Tumor histology (APA/PPA/SPA/Others)	0.002*	1.248	0.91-1.72	0.177
Tumor differentiation (Poor/Moderate/Well)	0.016*	0.498	0.31-0.81	0.005*
Lymph node metastasis (Present/Absent)	0.021*	2.363	0.90-6.20	0.081
Recurrence (Present/Absent)	0.383	0.731	0.36-1.47	0.381
Smoking history (Present/Absent)	0.053	1.167	0.62-2.21	0.635
Notch-1 expression (Positive/Negative)	0.033	0.540	0.29-1.02	0.057

RR: Relative risk, *P < 0.05.

## Discussion

LAD is highly heterogeneous, and its level of differentiation varies considerably. Sometimes, different parts of the same tumor showed distinct characteristics. In this research, the status of Notch-1 expression was observed to be associated with clinical stage, histological subtypes and survival outcomes of LAD patients.

Notch-1 was first found to associate with hematological diseases, and its expression level increased in multiple myeloma, Hodgkin’s lymphoma, anaplastic large cell lymphoma and acute myeloid leukemia [13,14]. Recently, Notch-1 was widely studied and reported to aberrantly express in malignant tumors [15-19]. It was considered as a highly controversial gene because of its complex biological functions. Some researchers demonstrated that the up-regulation of Notch receptors and ligands such as Notch-1 and Jagged-1 will probably predict relatively metastasis in lung cancer [20]. Notwithstanding that high expression of Notch-1 in a subgroup of NSCLC cells might be reported as a poor prognostic factor [9], different people hold different views. Zheng et al. found that overexpression of Notch-1 could substantially cause A549, a typical LAD cell line, to obtain cell cycle arrest and may suppress the growth of cancer [21]. Coincidentally, although Notch-1 may correlate with the prognosis of LAD patients in our study, its expression was also affected by other factors.

The binary properties of Notch-1 which behaves as either an oncogene or a tumor suppressor may possibly depend on the developmental stages or various histological types in LAD patients [21]. Herein, in view of the multidisciplinary classification of LAD, our data revealed that expression of Notch-1 is significantly correlated with histopathological subtypes of LAD. Some subtypes were easily got stained while others, particularly in SPA, were almost in a certain appearance of negative. On this basis, the prognosis of different histological types indicated significantly differences. Therefore, Notch-1 could be regulated by various factors during the development of LAD. Although the histologic heterogeneity is exactly an underlying complexity, we still consider that Notch-1 could serve as a meaningful biomarker for LAD patients. Maybe the expression linking with the subtypes is the reason why it acts as a protect factor in patients outcomes. Better survival has already been corroborated in LPAs, APAs and PPAs than in SPAs or MPAs, even though our selected cases contain much more of the former three types than the after. Probably, that’s the explanation of the survival analysis results of Notch-1 which was not in conformity with other literatures.

Interestingly, our results showed that the component of Notch signaling pathway is activated in both normal human alveolar or bronchial epithelium and lung tumor samples. It is unexpectedly that the level of Notch-1 protein was downregulated in LAD cells or tissues. The most reasonable explanation is what has been documented that Notch-1 could be trigged by hypoxia. Hypoxia acts as one of the major stimuli, the tumor microenvironment dramatically enhance Notch signaling in the progression of lung cancer, as well as many other types of tumorigenesis [22]. Expression levels of Notch signaling components in human lung cells, especially in primary bronchial epithelial and small airway epithelial, reflect observations in surgical specimens, yet lung tumor cell lines showed weakly positive, such as Notch-1. Chen’s results strengthen a strong nuclear staining for Notch-1 intracellular domain in lung epithelia, whereas adenocarcinoma samples manifested decreased NICD-1, even undetectable vision in some tumor areas. Nevertheless, hypoxia would dramatically activate the Notch signaling pathway in LAD cells, oxygen concertrations were contributed to regulate Notch activity in lung cancer [23]. Hypoxia may not only maintain malignant phenotypes of tumor cells but also cause poor response to treatment. This suggested that the functions of Notch pathway components in human LADs might be greatly influenced by tumor microenvironment.

Recently, it has been widely accepted that the dysregulation of the Notch signaling pathway existed in a variety of human tumors. Lung cancer has been characterized by a wide range of histological types. The heterogeneity of lung cancer, especially in NSCLC, had appeared obviously. Sometimes, a single one tumor may concurrently contain both adenocarcinoma behavior and squamous carcinoma performances. The differentiation may from startlingly well differentiated to entirely undifferentiated at the same time. As a receptor of Notch signaling pathway, Notch-1 was recommended as a vital factor in growth and development of various tumors. Some drugs which targeting the Notch signaling pathway has been taken into the clinical trials, used in the treatment of Alzheimer's disease and solid tumors [24,25]. Herein, our results demonstrated that the expression of Notch-1 was co-associated with histological types of LAD patients. Although Notch-1 could not be an independent prognostic factor, we propose that it would be a significant predictive indicator, which was used to differentiate histological type of LADs. Moreover, Notch-1 was actually a contradiction community. It could exert different biological functions which influenced by many unknown factors, and this need to be further studied.

All the possible reasons were verified by more and more researchers. The function of Notch-1 was also found to be required for tumor initiation via regulating P53 stability. The results of Licciulli implicated that Notch-1 was a pivotal effector in Kras-driven Lung adenocarcinoma and a critical P53 regulator at a posttranslational level [26]. Of interest, just like Kluk detected NICD1 staining in 151 NSCLCs, none of them showed diffuse strong staining. Thus, activation of Notch-1 doesn’t appear to be common in some solid tumors [27].

Taken together, downregulation of Notch-1 might be correlated with LAD development. Although Notch-1 was not an independent prognostic factor, it could be used as a predictable biomarker to be detected in different pathological and histological subtypes in LAD patients. Also, LAD patients with positive Notch-1 expression tend to have a prolong survival time. On the other hand, Notch-1 expression was figured out to associate with histological subtypes of LAD, which had totally disparate outcomes. Although further certification was needed, we still believe that the multiple roles of Notch-1 in NSCLC biology as well as its complex mechanisms should be further investigated in future.

## Abbreviations

LAD: Lung adenocarcinoma; NSCLC: Non-small cell lung cancer; IASLC/ATS/ERS: International Association for the Study of Lung Cancer/American Thoracic Society/European Respiratory Society; OS: Overall survival; ATCC: American type culture collection; FBS: Fetal bovine serum; DMEM: Dulbecco’s Modified Eagle’s Medium; RT-PCR: Reverse transcription-polymerase chain reaction; PMSF: Phenylmethanesulfonyl fluoride; SDS-PAGE: SDS- polyacrylamide gel electrophoresis; HRP: Horseradish peroxidase; H&E: Hematoxylin and eosin; PBS: Phosphate buffer solution; DAB: Diaminobenzidine; UICC: Union for International Cancer Control; APA: Acinar predominant adenocarcinoma; PPA: Papillary predominant adenocarcinoma; SPA: Solid predominant adenocarcinoma; LPA: Lepidic predominant adenocarcinoma; MPA: Micropapillary predominant adenocarcinoma; TTF-1: Thyroid transcription factor 1; NICD: Notch intracellular domain; IGF-1R: Insulin-like growth factor 1.

## Competing interests

The authors declare that they have no competing of interests.

## Authors' contributions

RW and LC conceived of the study, and participated in its designed. JH and HS participated in the experiments and drafted the manuscript. BL contributed to the sample collection and interpretation the data. JH performed the statistical analysis. BY carried out the immunohistochemistry. LC and RW revised the manuscript. All authors read and approved the final manuscript.
